# In memoriam: Professor Gerhard Malnic

**DOI:** 10.1590/2175-8239-JBN-e2023IM001en

**Published:** 2023-06-23

**Authors:** Nancy Amaral Rebouças, Roberto Zatz, Claudia Maria de Barros Helou

**Affiliations:** 1 Universidade de São Paulo, Instituto de Ciências Biomédicas, São Paulo, SP, Brazil.; 2 Faculdade Israelita Ciências da Saúde Albert Einstein, São Paulo, SP, Brazil.; 3 Universidade de São Paulo, Faculdade de Medicina, São Paulo, SP, Brazil.; 4 Hospital das Clínicas da Faculdade de Medicina da Universidade de São Paulo, Laboratório de Pesquisa Básica, LIM-12, São Paulo, SP, Brazil.

What can we say about Professor Gerhard Malnic?

First of all, we would like to mention some characteristics of his life, especially as a professor, followed by his notoriety in the scientific world in the field of renal physiology.

He obtained his medical degree in 1957 at the Faculdade de Medicina da Universidade de São Paulo (FMUSP). He then obtained a Doctor of Philosophy (PhD) degree from the same institution, where he studied renal chloride excretion under the supervision of Prof. Alberto Carvalho da Silva in 1960. He dedicated his entire professional life to this institution, where he both trained under-graduate and graduate students and conducted important scientific research.

**Figure 1.**
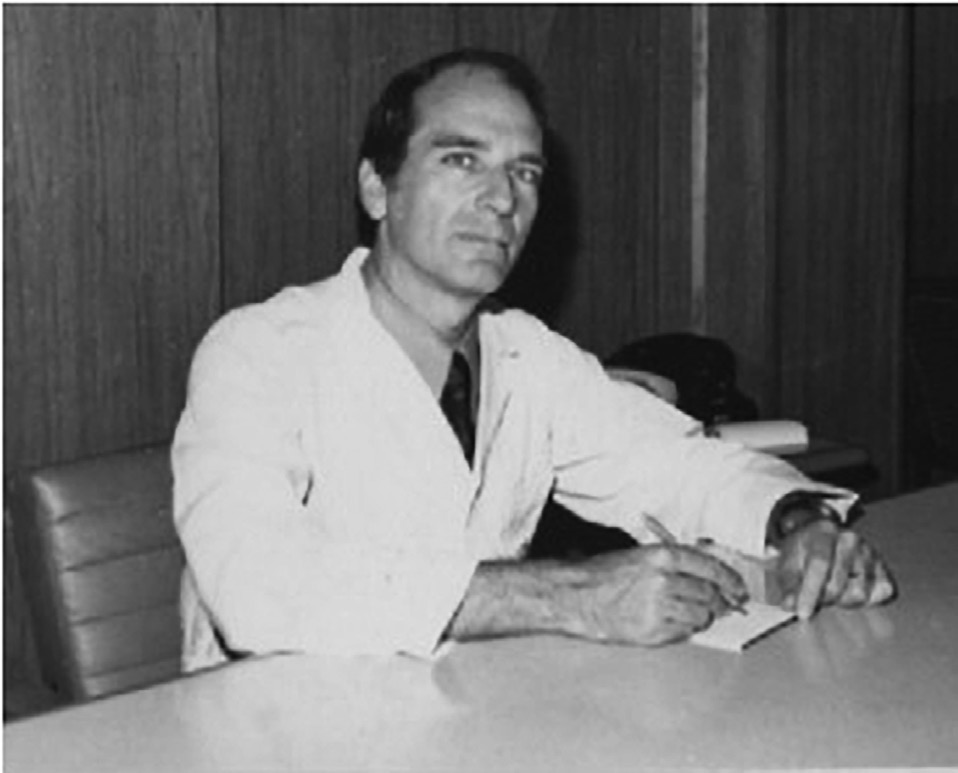
Professor Gerhard Malnic in his office in Departamento de Fisiologia do Instituto de Ciências Biomédicas da Universidade de São Paulo (ICB-USP), SP, Brasil in 1979.

Prof. Malnic was always present in the classrooms of the students of FMUSP and Instituto de Ciências Biomédicas (ICB) at USP, even after his retirement. He answered all kinds of questions with charisma and sympathy. Even with nonsensical questions, he always started the explanation with a gentle voice: “think about it, it’s not like that!”. This is one of the reasons why we often find his photo in the honored professors’ gallery in the photo album of many medical graduates of FMUSP (Figure 1).

Moreover, Prof. Malnic supervised several graduate students in their PhD thesis and he also contributed as a post-doctoral advisor of many students who needed more knowledge in the field of kidney physiology. Many of them are still working today as professors or researchers in remarkable and recognized Brazilian and foreign universities or research institutes. We would like to highlight Prof. Malnic’s collaboration in the experimental studies of his former students, until recently.

Prof. Nancy Rebouças is one of these students and she wrote the following lines:

I consider it a privilege to have had Prof. Malnic as my PhD supervisor, and also my office was right next to his at ICB-USP. Thus, I had a close professional relationship with him for at least 35 years. I performed several experiments with him and learned a lot about the biophysics of renal tubular transporters, especially during the development of our experimental research.

Another point I’d like to mention about Prof. Malnic is his interest in scientific advances in new methods and tools that contribute to a better understanding of renal physiology. When I decided to do post-doctoral studies abroad in the late 1980s, he suggested that I learn molecular biology methods that could be applied in our ICB research group when I returned to Brazil. Thus, we had molecular biology techniques combined with traditional electrophysiology, such as micropuncture or microperfusion in vivo, as well as intracellular determinations of both pH and Ca^2+^ concentration using fluorescent probes, whose quantifications were determined in images acquired by confocal microscopy in our new studies.

Having worked daily in Prof. Malnic’s team, I can mention other qualities of his personality that we all cherished. All the equipment in his laboratory was always open to us, including that which he had acquired with a special grant. He never demonstrated any controlling power. We used all the instruments and devices on our own responsibility without previous authorization. Likewise, we had his intellectual supervision whenever we needed it. He was truly a full professor who never competed with a former student or showed jealousy of a new investigation result that we could discover. On the contrary, he spurred us all on to reach ever higher ranks in our academic careers. His notoriety in the scientific community was decisive in all of his team members receiving FAPESP grants for their studies. We often collaborated with him, but actually he collaborated with us much more.

Every time we asked him for help, he was ready in a serious and responsible way. When I was preparing the questions for the student exam, I always wanted his opinion. I was sure that he would read the questions carefully and make suggestions to improve the exam when he thought it was necessary, and he never let a typo slip through. He contributed equally to teaching or on exams, even on weekends. He was always approachable and friendly to both professors and students at all levels.

Since I was in the renal physiology laboratory at ICB-USP every day, Prof. Malnic contributed a lot to a pleasant work environment. This feeling is also reported by the technician Camila and all students that I supervised. Almost all of them tell me that their time at ICB-USP was the best period of their lives!

In the following paragraphs, we would like to pay special attention to Prof. Zatz’s considerations about Prof. Malnic’s pioneering work in renal physiology. How he remarkably developed the experiments to demonstrate the mechanisms by which the renal tubules play an important role in electrolyte homeostasis in the body.

During his post-doctoral studies at Cornell University in the United States, Prof. Malnic made important contributions to the elucidation of potassium and hydrogen excretion in the kidney. His notable results about potassium renal excretion were published in the American Journal of Physiology^1,2^. He then returned to Brazil and continued his experiments to reveal the crucial mechanisms of how acids are excreted by the kidneys^3^. It is very important to mention that Prof. Malnic and his co-workers used complex techniques and manufactured many devices themselves to analyze microfluid collected from the kidneys under optical microscopy.

In the following decades, Prof. Malnic’s team developed a series of remarkable studies. Most of them allowed researchers to perform new evaluations to better understand kidney function and the effects of medications, such as diuretics.

Malnic’s studies on the role of the kidneys in potassium homeostasis, published in scientific journals in the 1960s are not outdated. These studies were critical in the discovery of K^+^ channels that are activated when fluid increases in the distal tubule, later known as the big K^+^ channels (BK) that were described in the early 1990s^4^.

In addition, Malnic’s studies also contributed to learning how H^+^ is processed in the renal tubules. His collaborations have been crucial to this day. We would like to point out the intratubular glucose effect on H^+^ secretion via the Na^+^/H^+^ antiporter (NHE3) action^5,6^. This knowledge is of great scientific interest today. The glucose-sodium cotransporter (SGLT2) is responsible for the reabsorption of these molecules in the proximal tubule. Inhibition of SGLT2 has been demonstrated to improve the renal function survival in patients with heart failure and metabolic syndrome and also slow the progression of chronic kidney disease.

Finally, we would like to mention Prof. Malnic’s contributions as a competent manager. He was Director of the Department of Physiology at ICB-USP from 1978 to 1981 and from 1984 to 1988. From 1983 to 1985 he was President of the Brazilian Society of Biophysics, the Brazilian Society of Physiology, and from 1995 to 1997 he was President of the Science Academia of São Paulo. Moreover, he was director of ICB-USP from 1989 to 1993 and of Instituto de Estudos Avançados at USP from 2001 to 2003.

Prof. Malnic received many awards, three of which are highlighted: 1) G.A. Borelli Medal, Frederico II University, Napoli, Italia in 1995; 2) Ordem Nacional do Mérito Científico, Comendador, Governo Federal do Brasil in 1995; and 3) Ordem Nacional do Mérito Científico, Grã-Cruz, Governo Federal do Brasil in 2000.
